# Beyond the Usual Suspects: *Weeksella virosa* as a Potential Human and Animal Pathogen

**DOI:** 10.3390/tropicalmed10080210

**Published:** 2025-07-26

**Authors:** Ioana Alina Colosi, Dan Alexandru Toc, Vlad Sever Neculicioiu, Paul-Ștefan Panaitescu, Pavel Șchiopu, Adrian-Gabriel Pană, Razvan Vlad Opris, Alina Mihaela Baciu, George Berar, Alexandru Botan, Carmen Costache

**Affiliations:** 1 Department of Microbiology, Iuliu Hatieganu University of Medicine and Pharmacy, 400349 Cluj-Napoca, Romania; 2 Faculty of Medicine, Iuliu Hatieganu University of Medicine and Pharmacy, 400012 Cluj-Napoca, Romania

**Keywords:** *Weeksellaceae*, Bacteriodota, Gram-negative bacteria, non-fermenting, opportunistic infections, healthcare associated infections, rare pathogens, 16S rRNA, MALDI-MS, antibiotic resistance

## Abstract

*Weeksella virosa* (*W. virosa*) is a rare, non-saccharolytic Gram-negative bacterium initially described in the 1970s, later proposed as a distinct genus in 1986. The genus *Weeksella* currently contains two species, namely *W. virosa* and *W. massiliensis.* Although primarily considered non-pathogenic, recent evidence has linked *W. virosa* to a limited number of clinical infections, mostly in immunocompromised patients. This review aims to consolidate the current body of knowledge on *W. virosa*, encompassing its microbiological and biochemical characteristics, involvement in human and animal infections, antimicrobial susceptibility profiles, and a critical evaluation of existing diagnostic methodologies. This review includes 13 case reports detailing 16 human cases retrieved from multiple databases, highlighting diagnostic inconsistencies and a lack of standardized antimicrobial susceptibility testing. Although *W. virosa* is generally susceptible to most antibiotics with the exception of aminoglycosides, recent reports seem to suggest a possible emerging resistance trend. The presence of this organism in hospital environments raises concerns about its potential transmission within healthcare settings. While biochemical testing appears to offer reasonably accurate identification of *W. virosa*, molecular confirmation may be warranted in some cases mainly due to the organism’s rarity. The reliability of MALDI-TOF MS for the identification of *W. virosa* remains currently uncertain. Further studies, including electron microscopy and genome-wide analysis, are urgently needed to clarify the pathogenic potential of this bacterium and guide clinical management. This review underscores the necessity for awareness among clinicians and microbiologists regarding this underrecognized pathogen.

## 1. Introduction

*Weeksella virosa* (*W. virosa*—formerly CDC group IIf) is a rarely encountered Gram-negative bacillus, historically classified within the *Flavobacteriaceae* family (order Flavobacteriales) and later proposed as a distinct genus by Holmes et al. in 1986 [1]. Currently, the genus *Weeksella* (family *Weeksellaceae*) encompasses two distinct species: *W. virosa* (Latin for “slimy”) and *Weeksella massiliensis* (Latin for “Marseille”) [2]. The initial strains of *W. virosa* were recovered mostly from genital tract secretions from women but also from various other clinical specimens such as urine, rectal swabs, cerebrospinal fluid, and eye and ear swabs, among others [1]. *W. virosa* has also been detected in the oral cavity [3], and further studies have identified the female genitourinary tract as an apparent significant reservoir, with an incidence of 0.42–2% in the general population and reaching 15% in some populations [4,5].

The clinical significance of *W. virosa* remains poorly understood. Initially considered to be non-pathogenic [6], a growing number of case reports have documented its involvement in human infections, in both immunocompetent [7] but mainly in immunocompromised patients [8,9,10]. The incidence of human *W. virosa* infections remains undetermined, most likely due to its taxonomic obscurity, limited awareness among both laboratory personnel and clinicians, and subsequent underreporting. The limited knowledge surrounding this bacterium is highlighted by the sparse and sometimes contradictory literature, with discrepancies noted even in fundamental phenotypic characteristics such as growth on MacConkey agar [11,12].

Although biochemical methods appear to provide reliable identification of this organism, advancements in molecular diagnostics—particularly 16S rRNA gene sequencing—have increasingly been utilized to confirm its identification with greater precision [13,14].

Given its environmental presence in hospital environments such as washbasins [15], water filtration devices [16], on apparel belonging to healthcare providers [17], and its potential for antibiotic resistance [14,18], a re-evaluation of *W. virosa* as a clinically relevant organism is warranted.

This review aims to consolidate both historical and recent knowledge on *W. virosa*, aiming to clarify its clinical significance in both human and animal infections, diagnostic challenges, antimicrobial susceptibility patterns, and treatment options.

## 2. *Weeksella virosa*—A Brief Taxonomic History and Emerging Genetic Perspectives

### 2.1. A Brief Taxonomic History of W. virosa

*W. virosa* was initially described by Pickett and Pendersen in 1970 [19]. Their study depicts three main groups of flavobacteria divided based on their resemblance to *Flavobacterium meningosepticum* (group one) and their saccharolytic activity. Among the non-saccharolytic group, eight strains could not be further identified at the time of analysis. One year later, Olsen and Ravn conducted a similar study in which they described twelve strains of non-saccharolytic flavobacteria with a different antibiotic susceptibility profile. Notably, these strains demonstrated the ability to grow at 42 °C, a characteristic atypical for flavobacteria [20]. Still, though they had data supporting the possibility of uncovering a new bacterium, they could not differentiate between *W. virosa* and other *Flavobacterium* species. Thus, those twelve strains remained classified as *Flavobacterium meningosepticum*. In the same time frame, with the help of Weaver and his personal communication, Owen and Snell managed to compare Weaver’s group (IIf) to both *Flavobacterium* and *Moraxella* [21]. After further research, they concluded that group IIf, which was similar to group three described by Pickett and Pendersen [19] and the group analyzed by Olsen and Raven [20], could not be part of either *Moraxella* or *Flavobacterium*. Finally, Owen and Holmes [6] divided the once unitary flavobacterium taxa into a saccharolytic and non-saccharolytic group. In these two main groups, five species were described: *Flavobacterium breve*, *Flavobacterium meningosepticum*, *Flavobacterium odoratum*, CDC Group IIb, CDC group IIk type 2, and lastly, CDC group IIf. Interestingly, this study reported that CDC group IIf did not cause disease in humans, in contrast to more recent findings. A recent study employing machine learning models to investigate the correlation between DNA biomarkers and pathogenicity classified *W. virosa* as non-pathogenic [22]. In 1986, Holmes et al. unified the research around flavobacterium using DNA analysis as well as biochemical and culture proprieties, finally naming the CDC group IIf as *Weeksella virosa* [1]. They have suggested naming the genus *Weeksella* after Prof O. B. Weeks [1] in order to honor his considerable research surrounding *Flavobacterium*. This study also compares other non-saccharolytic Gram-negative bacteria with similar Guanine + Cytosine (G + C) content to *W. virosa*, underlining the necessity of naming a new genus while better defining *Flavobacterium* which has been historically notorious for having loose criteria of inclusion [23]. Based on phylogenetic analyses, the relatively new *Weeksellaceae* family currently comprises several bacterial genera, including *Weeksella* (type genus), *Elizabethkingia*, *Chryseobacterium*, *Empedobacter*, *Bergeyella*, and *Wautersiella*, among several others [2].

### 2.2. Genomic Perspectives

The genome information of *W. virosa* remains relatively limited. The first genome sequence of *W. virosa* type strain 9751^T^ (DSM 16922—isolated from urine and described by Holmes et al. in 1986) was published in 2011. The complete genome was described as being ~2.3 Mb in size, with a GC content of 35.9%, containing 2181 genes of which more than 65% were annotated with a presumed function [12]. Currently, only six *W. virosa* genome entries are available in the NIH Genome database [24].

As previously discussed, based on early phylogenetic analyses, *W. virosa* was originally considered a close relative of both *Moraxella* and *Flavobacterium*. One of the closest phylogenetic relatives of *W. virosa* is represented by *Weeksella massiliensis*; strain FF8^T^ was isolated in Senegal from the urine of an elderly patient with acute cystitis and presented a 98.38% sequence similarity of 16S rRNA to *W. virosa* (2.5 Mb genome, GC content 35.9%) [2,25,26]. Another relatively recently described bacterial genus *Vaginella massiliensis* (type strain Marseille P2517^T^), isolated from the genital tract of a healthy woman, appears to share notable genetic similarity with *W. virosa*, exhibiting 93.03% 16sRNA sequence similarity; furthermore, *Vaginella massiliensis* has been found to possess a similarly sized genome as *W. virosa*, with nearly half of the orthologous proteins shared between the two genomes [27]. Other close phylogenetic relatives of *W. virosa* include *Chishuiella changwenlii*, *Empedobacter brevis*, *Moheibacter sediminis,* and *Algoriella xinjiangensis*; furthermore, *W. virosa* is also closely related to several *Chryseobacterium* and *Elizabethkingia* species (e.g., *Elizabethkingia meningoseptica*) [2,27]. Interestingly, the *OmpA/MotB* gene and protein from *Riemerella anatipestifer* seem to share a close evolutionary relationship with those from *W. virosa* DSM16922, *Elizabethkingia anophelis* Ag1, and *Flavobacteriaceae* bacterium 3519-10, possibly suggesting similar functions [28]. Another bacterium that seems to be more distantly related to *W. virosa* is *Aegyptianella ranarum*. Previous analyses revealed that although this organism shared only 81.2% genetic identity with *W. virosa*, it clustered within the same clade in the phylogenetic analysis [29].

## 3. *Weeksella virosa*: A Rare Pathogen in Humans

**Figure 1:**
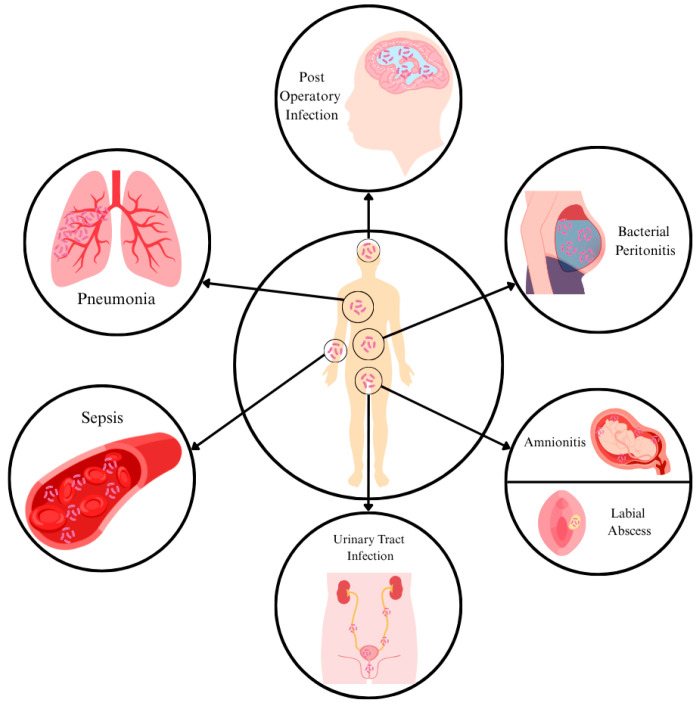
Case reports attributed to *Weeksella virosa.*

In order to evaluate the involvement of *W. virosa* in human infections, several broad searches were performed in Web of Science, PubMed, Embase, MedNar, Scopus, and the Cochrane Library for case reports and case series written in either English, Spanish, German, or French. To ensure comprehensive coverage of the available literature, we conducted searches across all aforementioned databases using various combinations of the following keywords: “Weeksella virosa”, “Group IIf”, “case report”, “case”, “infection”, “patient”, “AND”,“OR”. The precise search algorithm used in PubMed was the following: (“Weeksella virosa” OR Weeksella OR “Group IIf” OR Weeksellaceae) AND (case OR “case report” OR “case study” OR infection OR patient). Further searches of the gray literature were performed. The references of the included studies were manually screened, and additional relevant studies were subsequently included in this review. The searches yielded a total of 13 case reports, with one article describing 4 cases [8]. A synthesis of all included cases is presented in Table 1 and Figure 1.

To the best of our knowledge, we have reviewed all published articles concerning *W. virosa*, including those that explicitly reference the species by name as well as those describing a Gram-negative bacterium exhibiting the full set of characteristics attributed to *W. virosa*. Out of all the articles we have covered, there were 13 case reports of *W. virosa* with a total of 16 described human infections, the first one being described in 1991 [23].

In the outlined case reports, *W. virosa* has been recovered from a wide range of clinical samples, including peritoneal fluid, blood, wounds, urine, sputum, aqueous and vitreous samples, placenta, bronchoalveolar lavage, extradural and ventricular purulent material, and CSF. Consequently, *W. virosa* has been implicated in diverse types of infections, with the most common presentation being sepsis (*n* = 6/16, 37.5%) and peritonitis (*n* = 3/16, 18.75%). In all documented instances where patient data were available, affected individuals exhibited either significant comorbid conditions or immunodeficiency, suggesting a potentially limited pathogenic potential of this bacterium. However, Tamayo et al. reported a case in which an otherwise healthy adult female developed reticular lymphangitis following a dog bite [7]. Most of the reported cases demonstrated a favorable clinical course, with a relatively low associated mortality rate (*n* = 3/16, ~19%). Several antimicrobial regimens were described across the reported cases, with the majority involving various β-lactam antibiotics, administered either as monotherapy or in combination with other agents. Antimicrobial susceptibility testing results were only provided in half the cases (*n* = 8/16, 50%).

There seem to be no specific symptoms or signs associated with *W. virosa* infections. Moreover, the ability to produce a variety of infections in humans while also being discovered incidentally in the genitourinary tract of female patients with no symptomatology complicates clinical diagnosis due to its nonspecific clinical presentations [1].

Another noteworthy aspect is the detection of *W. virosa* in the surrounding environment. Although a limited number of studies address this topic, this bacterium has been found in water microfiltration devices, hospital washbasin taps, and the watches of healthcare workers. *W. virosa* appears to have a limited presence in the general environment, with its host interactions remaining unclear, as it may exist as a parasite, saprophyte, or commensal on various internal surfaces of humans and animals [36]. *W. virosa* has been recovered from point-of-use water filtration devices (1.8% and 2.4% of carbonated and chilled water samples) among other non-fermentative Gram-negatives bacteria, suggesting a potential risk, especially to vulnerable individuals [16]. Interestingly, one article reports the isolation of *W. virosa* strains from washbasin taps in hospital settings [15]. Although Staphylococci represent the most prevalent skin flora and are common contaminants of personal items, *W. virosa* has also been isolated from the wristwatch of an anesthesiologist [17]. To our knowledge, the potential human transmission of *W. virosa* in hospital settings has not been formally investigated to date. However, reported cases of healthcare-associated infections may point towards such a route of transmission [18,32]. Washbasin taps are recognized as potential reservoirs for various multidrug-resistant pathogens (e.g., *Pseudomonas aeruginosa* and *Enterobacter cloacae*) and have been implicated as sources of infection in healthcare environments [15,37]. Given the opportunistic nature of *W. virosa*, this may constitute a plausible route of transmission, particularly posing a risk to immunosuppressed individuals; however, further studies are required to confirm this.

## 4. Challenges in the Microbiological Diagnosis of *Weeksella virosa*

While the majority of both common and uncommon infections have well-established diagnostic and treatment guidelines, emerging or less-studied bacterial species often lack standardized approaches. Continuous research is essential to identify optimal diagnostic methods and therapeutic strategies. In the case of rare or poorly characterized pathogens such as *W. virosa*, clinical management is challenged by limited data regarding their pathogenic potential, disease progression, prognosis, and antibiotic susceptibility pattern.

### 4.1. Phenotypical Characteristics

The microbiological diagnosis of *W. virosa* can be challenging, as this bacterium exhibits limited particular microscopic or culture characteristics that might help with the preliminary diagnosis. *W. virosa* are nonmotile, strictly aerobic, non-spore-forming Gram-negative rods with a wide temperature growth range between 18 and 42 °C [36]. Colonies of *W. virosa* grown on various media (e.g., nutrient and blood agar) display an intensely mucoid cream-colored aspect [5,18,38]; however, it is worth noting that other sources describe them as non-pigmented [36]. One of the most frequently reported culture characteristics is the inability of *W. virosa* to grow on MacConkey agar [4,11,39]; however, this trait remains inconclusive, as conflicting results have been reported in the literature [12,36], suggesting phenotypical variability or methodological inconsistencies. While the presence of a capsule has not been formally described, it is heavily implied by the mucoid colony morphology.

The application of fluorescently labeled antibodies targeting bacterial components may represent a valuable approach for elucidating the structural characteristics of this bacterium. So far, there has been no study analyzing the structural components of *W. virosa* using fluorescence. Electron microscopy represents a critical tool for elucidating the structural features of *W. virosa*. To date, limited electron microscopy data are available regarding *W. virosa* [12]. Future studies employing this technique may provide valuable morphological insights that could further contribute to the development of more effective identification and treatment strategies.

### 4.2. Biochemical Characteristics

*W. virosa* is inert in most traditional biochemical tests. This organism is non-saccharolytic, oxidase and catalase positive, indole positive, urease negative, and has the ability to digest casein and hydrolyze gelatin [36]. Notably, several studies have reported that *W. virosa* yields a positive indole reaction with Ehrlich’s reagent, whereas the result is negative when tested with Kovac’s reagent [1,4,5]. Furthermore, *W. virosa* also appears to be pyrrolidonyl arylamidase (PYR) positive [40]. All other biochemical tests are indicative but not specific to *W. virosa* and thus cannot be used to make a microbiological diagnosis. Further studies analyzing the biochemical profile of this bacterium have shown relative consistency over the years regarding both the test used and the obtained results [4,5,12,14,26,27,29,40,41,42].

### 4.3. Laboratory Diagnosis of W. virosa

The primary challenge in the laboratory identification of *W. virosa* lies in its rarity and the limited awareness surrounding the organism. Currently, there is no consensus on the proper way to identify *W. virosa*; however, 16S rRNA gene sequencing has been suggested as the most indicated method for its identification [36]. Based on the included case reports, standardized culture followed by biochemical testing (including traditional biochemical tests, API panels, or automated approaches such as VITEK and BD Phoenix) seems to be a reliable way to identify this organism (Table 1). However, most likely due to its uncommon occurrence, molecular techniques have frequently been utilized for confirmatory identification, including 16S PCR and sequencing [8,14,18,31,32,34]. Some examples of primers used for this technique are as follows: Forward 5′-CGCTCGTTGCGGGACTTAACCCAACATCTC-3′, and reverse: 5′-GAGAGTTTGATCGTGGCTCAGATTGAACGC-3′ [8,14]. To date, cases in which *W. virosa* has been misidentified are sparse; however, to our knowledge, systematic comparisons of identification techniques are not available for this species. Given its genetic similarity to other flavobacteria, the possibility of misidentification by 16S rRNA PCR cannot be entirely excluded.

New diagnostic tools such as MALDI-TOF mass spectrometry are an excellent candidate for becoming the gold standard in correctly identifying rare pathogens, including Gram-negative non-fermenters. However, current evidence regarding the reliability of this method in the identification of *W. virosa* is conflicting and based on small sample sizes. One paper analyzing the efficacy of MALDI-TOF in the detection of non-fermentative Gram-negative bacilli has successfully managed to identify one *W. virosa* strain out of forty-eight other bacterial strains using this method [43]. Additionally, a comparative study of the Bruker Biotyper and BD Phoenix systems reported the successful identification of the sole *W. virosa* strain included in the analysis by both platforms [44]. Interestingly, a further recent comparison between the MALDI Biotyper and VITEKMS PRIME revealed that both platforms could correctly identify *W. virosa* [45]. Another study comparing the Bruker Biotyper with ASTA MicroIDSys highlighted a correct identification of *W. virosa* only in the case of the Bruker Biotyper [46]. Further limited evidence suggests that the MALDI Biotyper is capable of accurately identifying *W. virosa*, even in polymicrobial samples [47]. In contrast to previous reports, some evidence suggests that MALDI-TOF technology may be insufficiently reliable for the identification of *W. virosa*. Unalan et al. [14] reported a case in which *W. virosa* could not be identified using the Bruker MALDI Biotyper system; identification was initially achieved through biochemical profiling with the BD Phoenix system and subsequently confirmed via 16S rRNA PCR. In addition, one article evaluating the utility of direct pathogen identification from urine found MALDI-TOF to be unreliable in the diagnosis of *W. virosa* [48]. The outlined discrepancies further highlight the need for the formal validation of this technology.

In laboratory practice, ensuring diagnostic accuracy remains challenging. Despite advances in diagnostic tools and research, misdiagnosis can still occur due to equipment limitations, human error, or contamination, which, while preventable, is sometimes unavoidable. Limitations of standard biochemical testing are often evident in these cases, especially in regard to rare pathogens. In one reported case of peritoneal dialysis-associated peritonitis, *Dokdonella koreensis* was initially misidentified as *W. virosa* and *Brevundimonas* spp. by API 20NE and the VITEK 2 system. MALDI-TOF MS also failed to provide reliable identification. Definitive identification was ultimately achieved through 16S rRNA gene sequencing in this case [13].

## 5. Antibiotic Resistance and Treatment Challenges

Antimicrobial resistance (AMR) remains one of the most significant threats to public health at both local and global levels, with an estimated 1.14 million deaths directly attributable to resistant bacterial infections in 2021 [49]. In addition to the diagnostic challenges they present, rare and relatively rare pathogens also pose a significant threat due to their increased antimicrobial resistance [50]. Furthermore, antimicrobial treatments have been shown to alter complex bacterial communities in chronic infection models, leading to significant increases in several pathogens—including relatively rare species such as *Burkholderia cenocepacia* and *Achromobacter xylosoxidans* [51].

Other Gram-positive and Gram-negative bacteria display an alarming level of antibiotic resistance, with multiple genes and gene-regulating mechanisms being discovered at a never-before-seen rate. Close phylogenetic relatives of *Weeksella virosa*, such as *Elizabethkingia meningoseptica*, *Elizabethkingia anophelis*, and species within the *Chryseobacterium* genus, are well known for their broad intrinsic resistance profiles, particularly against most β-lactam antibiotics, among others [52]. Other relatively close phylogenetic relatives of *W. virosa,* such as *Flavobacterium,* display innate antibiotic resistance as well as environmentally acquired resistance [53]. Even studies looking at environmental freshwater microbiomes show that flavobacteria possess multiple resistances, with over 70% of isolates being classified as multiple-drug-resistant (MDR) in some cases [54].

As opposed to multiple related bacterial species, available data seem to suggest that *W. virosa* does not exhibit the same extent of antimicrobial resistance. To our knowledge, no intrinsic resistance to any antimicrobials has been formally validated in the literature regarding *W. virosa,* in contrast to *Bergeyella zoohelcum* (previously part of the genus *Weeksella*) which exhibits known resistance to polymyxin [55]. However, resistance to aminoglycosides has been previously suggested as a potentially inherent characteristic of this species [10]. In comparison, the only study describing a strain of *Weeksella massiliensis* highlighted a potential susceptibility to aminoglycosides (e.g., gentamycin) in this species [26]. At present, neither CLSI nor EUCAST provides interpretive breakpoints for antimicrobial susceptibility testing specific to *W. virosa* (CLSI M100 34th Edition and EUCAST v15.0). However, a limited interpretation of MIC values may be conducted following the provisions outlined by both guidelines for organisms lacking established breakpoints [56]. Currently, this remains the only validated approach for antimicrobial susceptibility testing of this species.

Based on previous data, it was generally accepted that *W. virosa* strains are most likely susceptible to both penicillin and also to most other antimicrobials [36]. Other data seem to confirm that *W. virosa* strains are susceptible to most beta lactams, chloramphenicol, and fluoroquinolones, while presenting variable MIC values to tetracycline and trimethoprim–sulfamethoxazole and resistance to one or several aminoglycosides. Notably, the combination of aminoglycoside resistance and penicillin susceptibility has been suggested as a potentially useful indicator for the identification of this organism [55]. The empirical use of piperacillin, aztreonam, and carbapenems, and avoiding trimethoprim–sulfamethoxazole, ciprofloxacin, and aminoglycosides in the absence of an antibiogram have been previously suggested [14,18].

Further studies examining the biochemical characteristics, antibiotic resistance profiles, and clinical case reports of *W. virosa* have yielded inconsistent findings regarding its susceptibility to antimicrobial agents; these inconsistencies may stem mostly from a lack of standardized testing, interpretation of the results, unclear reporting of AST, and due to underreporting of this organism. Based on the case reports in this review, the most commonly employed AST methods range from disk diffusion to MIC-based methods (macro- and microdilutions, automated methods such as VITEK, among others).

Various antimicrobial susceptibility profiles of *W. virosa* are available in the literature and are presented in Table 2. While the lack of standardization in testing methodologies and guidelines hinders extensive comparisons between most studies, *W. virosa* isolates with multiple antimicrobial resistances have been reported in recent years, in various countries: Turkey, the USA, and Mexico. Unalan et al. [14] reported a strain of *W. virosa* isolated from the peritoneal fluid of a 4-year-old female patient, exhibiting resistance to multiple cephalosporins, including ceftazidime, cefotaxime, and cefepime. The isolate was also categorized as intermediate to ciprofloxacin and amikacin based on CLSI interpretive criteria. Campbell et al. [34] describe a case of sepsis from an extremely low-weight premature infant determined by an isolate of *W. virosa,* which showed resistance to ceftriaxone and cefepime; however, the isolate was susceptible to ampicillin and meropenem. Of particular concern is a strain reported by de la Fuente García Peña et al. [18] isolated from bronchoalveolar lavage in a 64-year-old female with pneumonia. This strain exhibited resistance to most tested antimicrobials (including all tested beta lactams) and was only susceptible to ciprofloxacin. Based on the limited available evidence, these results might point towards the emergence of resistance in this bacterium and the need for ongoing surveillance.

In one study looking at healthcare-associated infections with Gram-negative bacteria, out of the 227 isolates from the 130 patients included in the study, *W. virosa* occurred in 0.4% of cases [57]. Even though infections with this bacterium are rare, the prevalence of *W. virosa* in the genitourinary tract of asymptomatic female patients is an important factor to consider when talking about the development of multiple antibiotic resistance. In the case of the misuse of antibiotics in urinary tract infections, even though the infection alongside the causing pathogen might be treated, there is still a risk of the local microflora, including but not limited to *W. virosa*, developing antibiotic resistance.

Due to the decline in both the discovery and engineering of new antibiotics, combined with the alarming rate in which bacteria develop resistances, it is essential to divert our attention from conventional treatment plans and start looking towards newer technology. Two of the most promising alternatives are bacteriophage therapy and CRISPR (clustered regularly interspaced short palindromic repeats) genetic engineering. Though controversial, they are the most effective and with the most promising results out of all other non-conventional options. Phage therapy represents a unique tool in fighting infections using viruses that target bacteria. Recently, several studies have shown promise in using phage therapy against resistant strains of *Klebsiella pneumoniae* and *Acinetobacter banumanii*; the main issue with this technique is finding a proper phage cocktail that targets the bacteria of interest [58,59,60]. Limited clinical trials using phage therapy were described, and the results were inconclusive [59,60]. CRISPR-Cas technology provides a unique gene editing tool to better tackle the ongoing fight against antimicrobial resistance [61,62]. In bacterial cells, the CRISPR system is used to edit specific sequences of interest. Studies have shown that CRISPR-Cas defective cells were able to accumulate several antimicrobial resistance genes; thus, reactivating this system might help to eliminate these genes. Studies have shown that CRISPR-Cas-deficient cells can accumulate multiple antimicrobial resistance genes [61,62]. Therefore, reactivating the CRISPR-Cas system could potentially help eliminate these genes. To our knowledge, no studies have explored the use of phage therapy or CRISPR-Cas technology to combat *W. virosa* infections yet.

**Table 2:** Antimicrobial susceptibility profiles of *W. virosa* isolates.

Reference/Antibiotic	Mardy et al., 1988 [4]	Reina et al., 1990 [5]	Fass et al., 1996 [63]	Tamayo et al., 2003 [7]	Manogaran et al., 2004 [10]	Slenker et al., 2012 [8]	Toescu et al., 2017 [32]	Unalan et al., 2019 [14]	Vaquera-Aparicio et al., 2020 [33]	Campbell et al., 2020 [34]	de la Fuente García Peña et al., 2024 [18]
Number of isolates (n)	n = 6	n = 3	n = 8	n = 1	n = 1	n = 1	n = 1	n = 1	n = 1	n = 1	n = 1
AST technique	DD	MIC	MIC	DD	n/a	MIC	n/a	MIC	MIC	MIC	DD and MIC
Penicillin	S										
Ampicillin	S	S ^		R					S	S	
Ampicillin–Sulbactam			S								R
Amoxicillin							S				
Amoxicillin–Clavulanic Acid							S				
Carbenicillin		S									
Ticarcillin		S									
Azlocillin		S									
Mezlocillin		S									
Piperacillin		S	S		S	S		S			
Piperacillin–Tazobactam			S				S		S		R
Cefazolin		S					#				
Cefuroxime		S					#		S		
Cefoxitin		S					#				R
Cefotaxime		S		R			#	R			
Ceftriaxone		S	S	S	S		#			R	R
Ceftazidime		S	S	S	S	S	#	R		I	R
Cefoperazone			S				#				
Cefepime					S		#	R		R	R
Aztreonam		S		S		S					
Imipenem		S	S	R	S ^^	S ^^		S			R
Meropenem						S	S	S		S	R
Ertapenem											R
Doripenem											R
Streptomycin	S										
Neomycin	R										
Kanamycin	R			R							
Gentamicin		R		R	R	S	R		S		R
Tobramycin		R	R *		R	R					
Amikacin		R		R		R		I	S		R
Nalidixic Acid		R		R							
Norfloxacin		S									
Ciprofloxacin		S	S	R	R	R	R	I	S		S
Ofloxacin			S								
Levofloxacin					R						
Tetracycline	S	R		S					S		
Tigecycline											I
Trimethoprim	R										
Trimethoprim–Sulfamethoxazole		R	I **	S	R						
Erythromycin	S										
Polymyxin B											
Colistin	S										
Cetrimide											
Nitrofurantoin		R									
Chloramphenicol	S	S		S							
Novobiocin	S										

Abbreviations: S—susceptible; R—resistant; I—intermediate resistant; * —13% of strains tested I; ** —75% of strains tested I and 25% of strains tested S; ^ combined with clavulanic acid; ^^ combined with cilstatin; #—S to unspecified cephalosporins; DD—disk diffusion; MIC—minimum inhibitory concentration based methods including broth macro- or microdilution, gradient test, and automated methods (Vitek, Sensititre ARIS 2X ID/AST, MicroScan Gram-Negative Combo panel).

## 6. *Weeksella virosa* Infections and Isolation in Animals

Although the main focus of this review remains the understanding, diagnosis, and treatment of *W. virosa* in human infections, investigating its impact on other animal species remains equally important. Such insights are not only valuable for veterinary medicine but also have significant implications for the agricultural sector and international trade. Moreover, elucidating the modes of transmission among animals—and the potential for zoonotic transfer to humans—remains a critical area of inquiry.

**Figure 2:**
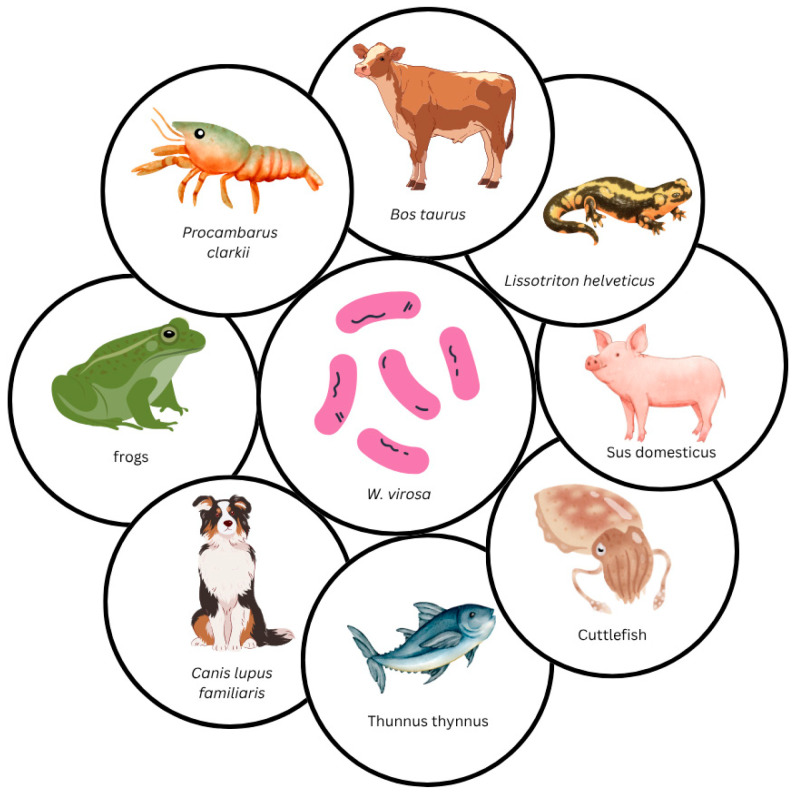
*Weeksella virosa*—from commensal to pathogen in animal hosts.

Animal species in which *W. virosa* has been identified or implicated in infections include cattle (*Bos taurus*) [64], dogs (*Canis lupus familiaris*) [65], crayfish (*Procambarus clarkii*) [42], cuttlefish (class *Cephalopoda*, subclass *Coleoidea*) [66], Atlantic bluefin tuna (*Thunnus thynnus*) [67], the Titicaca Lake forg (*Telmatobius coleus*) [68], domestic pigs (*Sus domesticus*) [69], tree frogs (*Hyla crepitans*) [70], and palmate newts (*Lissotriton helveticus*) [71]. The following section provides a concise overview of the relevant studies and their findings regarding the association between these animal species and *W. virosa* in the context of its uncertain pathogenic role, ranging from a potential pathogen to a commensal organism. An overview of these cases is presented in Figure 2.

Hamon et al. describe a case of nasal leiomyoma in a dog (*Canis lupus familiaris*) presenting with sneezing and epistaxis [65]. The tumor was diagnosed via CT and biopsy and was resected shortly after diagnosis. To prevent bleeding postoperatively, intranasal gauze was used. Following microbiological analysis, *W. virosa* was isolated from the gauze; however, the authors did not specify the diagnostic methods employed to identify the organism. They were treated with amoxicillin, clavulanic acid, and meloxicam to prevent a possible infection with this etiologic agent. At the 14-month follow-up, the CT scan revealed no tumor masses left. It is important to take into consideration the possibility of contamination of the gauze with commensal bacteria as well as contamination from other sources. Also, while a microbiologic diagnosis was provided, the lack of details surrounding this diagnosis raises questions regarding the accuracy and certitude with which this diagnosis was reached.

During the necropsy of a 7-year-old cow (*Bos taurus*), Brun et al. discovered a mass in the cow’s urinary bladder [64]. Subsequent histopathological examination revealed an infiltrative tumor that was confirmed to be a lymphoepithelioma-like carcinoma (LELCA) displaying cytokeratin at immunohistochemistry. A microbiological examination of the cancerous cells revealed high levels of bovine-papillomavirus type 2. Urine samples were taken from the deceased cow which yielded, among other bacteria, *W. virosa*. This diagnosis was reached using both the BBL^TM^ Crystal^TM^ kit and Slidex Staph Plus^TM^ kit. While useful, these testing methods do not correlate with the ones used for human infections. In addition, these diagnostic tools could be seen as insufficient to reach such a rare diagnosis.

Rich et al. conducted a retrospective study on pathological findings in captive cephalopods [66]. Sepsis associated with ulcerative dermatitis, gill, and digestive gland was among the most frequently reported infections in cuttlefish, with *W. virosa* identified among the associated bacterial pathogens; while identified, *W. virosa* was reported as a probable contaminant associated with spoilage. Similarly to the first discussed article, the diagnostic methods were not mentioned. Moreover, this article highlights the often overlooked issue of potential contamination, a concern rarely addressed in the other included studies. Similarly, *W. virosa/Empedobacter brevis* has also been potentially recovered from the skin swabs of clinically healthy Atlantic bluefin tuna (*Thunnus thynnus*) only during the spring and identified through API testing [67]. Another study analyzed the histopathology and microbiology of various species of crayfish from England [42]. Other bacterial species and *W. virosa* were detected on non-native crayfish, posing a potential risk for future infectious outbreaks in the native fauna. In this case, *W. virosa* was identified using API 20NE.

Another study mentions the isolation of bacteria from the genus *Weeksella* from swine lung samples in pigs with pneumonia lesions; however, in this case, the bacteria were identified through biochemical testing, not identified at a species level, and recovered in mixed culture with *Aerococcus suis* [69].

The Lake Titicaca frog (*Telmatobius culeus*) is a remarkable yet critically endangered species. Its population has declined significantly due to environmental pollution, invasive species, and poaching, placing it at high risk of extinction. To advance the understanding and conservation of this species, a study was conducted on the cutaneous microbiota of 14 Lake Titicaca frogs [68]. Using API 20E and API20NE biochemical tests, various bacterial strains were identified, including *W. virosa*. This article also highlights the possibility of human infections after contact with this species of frog, alluding even to infections with *Vibrio cholerae*.

One study focusing on the characterization of the phylogenetic tree of *Aegyptianella ranarum* has also observed that this bacterium parasitizes the erythrocytes of frogs [29]. While only distantly phylogenetically related, *W. virosa* could also potentially play a pathogenic role in frogs. Interestingly, one study identified *W. virosa,* through API 20E and API 20NE, as the sole bacterium in epidermal cysts of a tree frog (*Hyla crepitans*), indicating that it may be able to colonize cysts and determine epithelial necrosis [70].

Lastly, *W. virosa* has also been isolated from palmate newts (*Lissotriton helveticus*) from France and identified through API 20NE. In this case, *W. virosa* was interpreted as an environmental or commensal organism [71].

## 7. Limitations and Future Directions

This review highlights several limitations in regard to our knowledge of *W. virosa*. The underuse of molecular identification techniques underscores the potential for misidentification in the case of rare or unusual pathogens, including *W. virosa*. Additionally, most reported cases lack detailed identification protocols and standardized antibiotic susceptibility testing, with unclear interpretation guidelines. The pathogenic role of *W. virosa* remains uncertain in some cases, further complicating clinical relevance and treatment strategies.

Currently, there are several gaps in our understanding of *W. virosa*. Fundamental microbiological characteristics of this bacterium remain insufficiently defined, including its virulence factors and the potential presence of a capsule. Future studies should focus on validating commonly used diagnostic systems such as MALDI-TOF for reliable identification. Standardized protocols for antibiotic susceptibility testing and interpretation are also urgently needed. Additionally, clarifying the pathogenic potential of *W. virosa* through experimental studies will be essential to better understand its role in human and animal infections.

## 8. Conclusions

*Weeksella virosa* is an underrecognized, non-fermentative Gram-negative bacterium, historically considered non-pathogenic but now increasingly associated with opportunistic infections in humans. Our review identified 16 documented human cases in which *W. virosa* was associated with a diverse spectrum of infections, including peritonitis, sepsis, urinary tract infections, pneumonia, and postoperative complications, among others. In the majority of cases, immunosuppression or underlying comorbidities were present, suggesting that this bacterium may possess limited intrinsic pathogenic potential. Furthermore, one case of wound infection following a dog bite may suggest zoonotic potential. Environmental detection of *W. virosa* remains largely unexplored; however, it was recovered from various healthcare-associated settings (water filtration devices, hospital washbasin taps, and watches of healthcare workers).

Veterinary data—though limited—reveal the presence and pathogenic potential of *W. virosa* in diverse animal species. While pathogenicity remains uncertain in many of these cases, the repeated isolation from a wide range of both healthy and diseased animals may suggest a broader ecological role and possible zoonotic potential.

Diagnostic accuracy is hindered by the rare isolation of this bacterium. Biochemical testing seems to provide a sufficiently accurate identification; however, confirmation through molecular methods may be warranted in some cases. Data regarding MALDI-TOF MS are mostly conflicting and require further formal validation. Despite relatively low antibiotic resistance so far, variability in testing methods and emerging resistant strains underscore the need for standardization. Further molecular, epidemiological, and ultrastructural studies are essential to clarify the organism’s pathogenic capacity, refine diagnostic algorithms, and guide evidence-based therapeutic strategies.

*W. virosa* should be considered—particularly in immunocompromised patients—when a Gram-negative rod is isolated that demonstrates mucoid, pigmented colony morphology, no growth on MacConkey agar, and is positive for oxidase, catalase, and indole. Given the available evidence, resistance to aminoglycosides should be presumed during initial antimicrobial susceptibility assessment.

## Figures and Tables

**Table 1:** *Weeksella virosa* human case reports.

No	Reference	Patient Sex and Age (years)	Comorbidities/Risk Factors	Type of Infection	Sample	Identification Method	AST	Treatment	Outcome
1	Faber et al., 1991 [11]	F, 33	End-stage renal failure with peritoneal dialysis	Spontaneous bacterial peritonitis	Peritoneal fluid	n/a	n/a	IMP/Cilastin + AMP	Favorable
2	Boixeda et al., 1998 [9]	M, 55	HCV cirrhosis	Spontaneous bacterial peritonitis	Peritoneal fluid	VITEK GNI and API 20E	n/a	FOX	Favorable
3	Meharwal et al., 2002 [30]	n/a	n/a	UTI	Urine	n/a	n/a	n/a	n/a
4	Tamayo et al., 2003 [7]	F, 31	Immunocompetent	Reticular lymphangitis	Wound	Biochemical	Yes	TET	Favorable
5	Manogaran et al., 2004 [10]	F, 53	Lymphoma, chronic kidney failure with hemodialysis, diabetes mellitus	Pneumonia and sepsis	Sputum and blood	n/a	Yes	FEP + AB	Patient expired
6	Melo et al., 2011 [31]	n/a, 50	n/a	Postoperative infectious endophthalmitis	Aqueous and vitreous samples	Biochemical testing (Phoenix system) and real-time PCR	n/a	VAN + CTZ	Favorable
7	Slenker et al., 2012 [8]	F, 44	Obesity, menorrhagia	Labial abscess	Wound	n/a	n/a	Surgical	Favorable
		F, 26	Endometriosis, pelvic adhesions, small bowel obstruction, abdominal surgery, diabetes mellitus	UTI	Urine	n/a	n/a	SXT	Favorable
		F, 25	Pregnancy and vaginal delivery complicated by Amnionitis	Amnionitis	Placenta	n/a	n/a	AMP + CN	Favorable
		F, 31	Ischemic heart disease, acute myocardial infarction, end-stage renal disease with hemodialysis, smoking, asthma, HCV infection, obesity	Sepsis and suspected pneumonia	Blood	BD Phoenix and 16S gene sequencing	Yes	AZT + TOB + D	Patient expired
8	Toescu et al., 2017 [32]	F, 49	Glucocorticoid use, recurrent malignant meningioma treated with repeated surgeries and whole-brain radiotherapy	Sepsis due to post-surgical ventricular empyema	Extradural and ventricular purulent material, CSF, other tissue samples	16S rRNA PCR	Yes	CTR + AMX	Favorable (patient expired due to neoplastic complications)
9	Unalan et al., 2019 [14]	F, 4	Addison’s disease, terminal kidney failure with peritoneal dialysis	Bacterial peritonitis associated with peritoneal dialysis	Peritoneal and dialysis fluid	BD Phoenix and 16S gene sequencing	Yes	FEP, later MEM and catheter removal	Favorable
10	Vaquera-Aparicio et al., 2020 [33]	M, 4	Embryonal rhabdomyosarcoma	Bacteremia	Blood	Sensititre™ ARIS™ 2X ID/AST System-Thermo Fisher Scientific	Yes	IMP/cilstatin, MEM *	Favorable
11	Campbell et al., 2020 [34]	n/a, 26 weeks	Extremely premature with low birth weight	Neonatal Sepsis	Blood	16S rRNA PCR	Yes	MEM **	Favorable
12	de la Fuente García Peña et al., 2024 [18]	F, 64	Diabetes mellitus, chronic renal disease, transcatheter aortic valve implantation, post-cardiac arrest syndrome, mechanical ventilation, soft tissue infection in pelvic limb	Ventilator-associated pneumonia	Bronchoalveolar lavage	VITEK and PCR sequencing	Yes	CIP	Favorable
13	Dilip et al., 2024 [35]	M, 69	Chronic urinary catheterization, hypertension, hyperlipidemia, benign prostatic hyperplasia	Septic shock	Blood and urine	n/a	n/a	FEP	Patient expired

Abbreviations: n/a—data not available; M—male; F—female; HCV—Hepatitis C Virus; IMP—imipenem; AMP—ampicillin; FOX—cefoxitin; AB—amphotericin B; SXT—Trimethoprim Sulfamethoxazole; CN—gentamicin; AZT—azithromycin; TOB—tobramycin; D—Daptomycin; CTR—ceftriaxone; AMX—amoxicillin; FEP—cefepime; MEM—meropenem; CIP—ciprofloxacin; VAN—vancomycin; CTZ—ceftazidime; TET—tetracycline; PCR—polymerase chain reaction; UTI—Urinary tract infection; AST—antimicrobial susceptibility testing; * outpatient treatment with CIP; ** previously the patient was treated with AMP + CN and VAN + FEP.

## Data Availability

All data are available withing the article.
